# Introduction of “MAPS” wound healing index and its correlation with guided bone regeneration outcome

**DOI:** 10.1371/journal.pone.0319271

**Published:** 2025-03-20

**Authors:** Amanda Rodriguez, Diego Velasquez, Leonardo Marquez, Jose Maria Ramos, Nataly Zambrana, Maria Masotti, Oliver Kripfgans, Hsun-Liang Chan

**Affiliations:** 1 Department of Periodontics and Oral Medicine, School of Dentistry, University of Michigan, Ann Arbor, Michigan, United States of America; 2 Department of Periodontology, University of Illinois Chicago College of Dentistry, Chicago, Illinois, United States of America; 3 Private Practice, Fenton, Michigan, United States of America; 4 School of Dentistry, University of Michigan, Ann Arbor, Michigan, United States of America; 5 School of Public Health, University of Michigan, Ann Arbor, Michigan, United States of America; 6 Department of Radiology Michigan Medicine, University of Michigan, Ann Arbor, Michigan, United States of America; 7 Division of Periodontology, The Ohio State University College of Dentistry, Columbus, Ohio, United States of America; International Medical University, MALAYSIA

## Abstract

This study aims to introduce a new index that could become a framework for future modification and improvement, and retrospectively test the predictability of this index collectively and individually for final bone changes by using existing research data pertinent to guided bone regeneration (GBR).

**Methods:**

The MAPS score was introduced to evaluate the bioMechanical, Aesthetic/Anatomical, Pathophysiologic, and Subject-related parameters for the healing assessment of 20 patients who underwent GBR in the posterior mandible retrospectively. Intraoral photography was taken at 3-, 10-, 21 days, and 5 months, resulting in 80 follow-up visits. Two independent examiners evaluated the photos giving scores for each timepoint and tested against horizontal bone gain (CBCT) for predictability.

**Results:**

Cohen’s Kappa values showed high intra- and inter-examiner agreement. Pearson’s correlation showed an inverse correlation between baseline bone width and bone changes at a 3 mm level (R^2^ = 0.23). The higher M, A, and P values at any time point were associated with higher bone gain. The 10-day MAPS score turns out the most predictive of bone gain (RMSE 1.32, R^2^ 0.75). In addition, increasing the average P score by 1 point at 10 days is associated with an increase in bone gain of 1.23 (p=.057).

**Conclusion:**

The MAPS score improves consistently over the 5-month healing period. However, no statistically significant difference is observed between the scores at 21 days and 5 months, reflecting the clinical healing pattern for GBR. The overall MAPS score correlated with bone changes after GBR procedures, indicating its potential for estimating hard tissue regenerative outcomes.

## 1. Introduction

Wound healing monitoring after oral surgeries is crucial to ensure the recovery is on the right track [1,2]. Several synchronized biological events occur during the healing, including hemostasis, inflammation, proliferation, wound remodeling, and maturation [1,3]. In these intricate wound healing phases distinct types of cells, cytokines, growth factors, and extracellular matrix components participate at certain times and duration [1,2,4]. Deviation from the normal course results in a variety of clinical presentations and surgical outcomes [2,5–10]. Clinically, different indices have been proposed for periodontal wound healing as a result of soft and hard tissue surgical procedures [1,3,5,6,8,9,11–22]. Although these indices are different in aspects of the parameters used, the scoring mechanism, complexity, and the evaluation timing, by dissecting them there seem to be common themes that encircle around these healing phases [1,3,6,8,11,12,7–28]. Identifying and applying the key common parameters that already exist among the currently available indices could improve our understanding of wound healing evaluation.

Guided bone regeneration (GBR) is a surgical procedure performed to preserve and/or increase alveolar bone volume for proper implant function and esthetics [24,29–38]. Clinical and histologic evidence of its effectiveness has been demonstrated [29,35,39,40]. Yet a desirable outcome is in part guarded by the systemic conditions of the patient, hard and soft tissue characteristics, biomaterial selections, and clinician’s experience [24,33,37,38,41–44]. Complications may arise leading to inferior bone gain, including soft tissue lesions such as dehiscence, membrane exposure, tissue necrosis, acute infection, abscess, and wound opening [24,37,38,41]. These complications may appear more frequently in patients with systemic and behavioral risk factors, such as smoking history and uncontrolled diabetes [13–15,32,33,35–38,41–43,45–47]. When the surgical sites remain undisturbed during the initial healing phase, regenerative success will have a higher chance to occur. An uneventful soft tissue healing maintains the stability of the underneath biomaterial, nourishes the surgical site, and forms a protective barrier against biological and mechanical irritation [24,33,35,37,38,41,46,47]. Since GBR is an invasive, and expensive procedure with a long healing time, timely and effective wound evaluation and management could avoid or mitigate potential negative outcomes so as to benefit the patients.

Soft tissue dehiscence and membrane exposure are common after GBR, accounting for approximately 20% of the cases [24]. Significantly less bone gain was found with membrane exposure [37,47,48]. A classification of postoperative complications in GBR by using a non-resorbable membrane was presented ([49]. It is based on the size of the membrane exposure, presence of purulent exudate and abscess formation. Other clinical signs and symptoms that may be related to the healing status and ultimately the amount of bone augmentation should also be systematically evaluated. Given the growing application of GBR and a need for a better understanding of GBR wound healing, this study aims to (1) introduce a new index that could become a framework for future modification and improvement, and (2) retrospectively test the predictability of this index collectively and individually for final bone changes by using existing research data pertinent to GBR procedures.

## 2. Materials and methods

The study was approved by the University of Michigan Medical School Institutional Review Board (IRB) (HUM00161016) in accordance with the Helsinki Declaration of 1975, as revised in 2013. A written informed consent was obtained from all participants before their inclusion in the study. The recruitment period was from 7/23/2021 to 5/4/2023.

### 2.1. Introduction of the “MAPS” oral wound healing index

**Fig 1:**
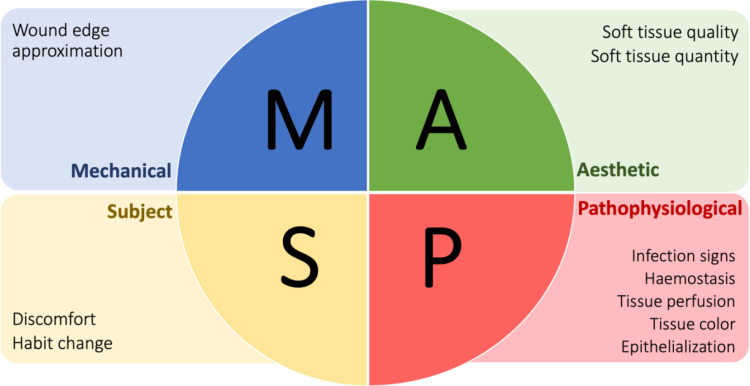
The “MAPS” Wound Healing Index. A comprehensive framework for categorizing the key parameters of wound healing.

Previous wound healing indices have identified some overlapping but distinct key parameters that are essential for assessing wound healing outcomes [1,3,9,11,7,17,19–22,50,51]. To sort these parameters and evaluate their collective as well as relative correlation to the guided bone regeneration outcomes, the “MAPS” wound healing index is herein introduced. The acronym “MAPS” stands for “bioMechanical, Aesthetic/Anatomical, Pathophysiologic, and Subject-related,” as illustrated in Fig 1. These four domains serve as a comprehensive framework for categorizing the key clinical parameters that are significantly related to wound healing. Within each domain, points are given based on a scale of 1 (compromised), 2 (questionable), 3 (normal), to 4 (optimal) for the evaluated parameters, as outlined in Table 1.

**Table 1:** MAPS Oral Wound Healing Index Quantification.

Category	Parameter	Compromise (Score of 1)	Questionable (Score of 2)	Normal (Score of 3)	Optimal (Score of 4)
**Mechanical**	Wound edge approximation	Open wound margins in full soft tissue thickness	Open wound margins but not full thickness	Edge-to-edge margin contact with visible incision line	Merged margins without an incision line
**Aesthetics**	Quality (discrepancy from adjacent tissues)	Major	Moderate	Minor	No
	Quantity (discrepancy from adjacent tissues)	Major	Moderate	Minor	No
**Physiological**	Signs of infection	Spontaneous suppuration or on light palpation	Obvious exudate on light palpation	Slight exudate under compression	No exudate
	Hemostasis	Spontaneous bleeding	Bleeding at incision margins	Presence of fibrin at incision margins	Absence of fibrin
	Tissue Perfusion	Entire flap necrosis	Partial soft tissue necrosis	Necrosis at focal non- critical site	No necrotic tissue and adequate perfusion
	Tissue Color Discoloration	Significant	Moderate	Slight	Normal
	Epithelialization	Absent	Partially present	Full with evidence of depression	Complete without depression
**Subject**	Discomfort	Severe	Moderate	Slight	Absent
	Habit change/Function impairment	Significant	Moderate	Slight	Absent

#### 2.1.1. Biomechanical domain.

This domain assesses wound edge approximation influenced by the biomechanics of the wound, e.g., residual flap tension, muscle pull, suture tightness, biomaterial stability, etc., during the healing time. The 4-point scale is proposed as:

- 1 point: an open wound margin through the full soft tissue thickness. The underlying biomaterials can be felt or seen up on light separation of the margin.
- 2 points: an open wound margin but not through the full thickness. The underlying biomaterials are not felt or seen.
- 3 points: edge-to-edge margin contacts but with a visible incision line.
- 4 points: merged margins with disappearance of the incision line.

#### 2.1.2. Aesthetic/Anatomical domain.

Wound healing can and has been assessed by the esthetic outcome, reflected by the degree of soft tissue harmony of the surgical site compared to adjacent sites. Soft tissue quality and quantity are evaluated separately. Soft tissue quality that impacts esthetics may include the presence of keloid, inadequate keratinized mucosa, and color changes. Soft tissue quantity that compromises esthetics may include loss of interdental papilla, facial tissue height, and overall tissue volume. To define the degree of discrepancy as:

- 1. Major: Significant differences in tissue quality and/or quantity, with noticeable changes in texture (presence of keloid), inadequate keratinized mucosa, color, or contour at the surgical site compared to adjacent sites.
- 2. Moderate: Some visible differences in tissue quality or quantity, but not as pronounced as in the major discrepancy category. Minor changes may be noted in texture, color, or contour.
- 3. Minor: Subtle differences in the quality or quantity of soft tissue, with only slight deviations from adjacent areas, often not easily noticeable under normal conditions.
- 4. No: The soft tissue at the surgical site appears identical to that of the adjacent sites, with no visible differences in texture, color, contour, or keratinized mucosa band.

In cases not in the esthetic zone, the soft tissue anatomical changes related to the quality and quantity after the surgery is evaluated.

#### 2.1.3. Pathophysiological domain.

This domain relates to host reactions in response to trauma from the surgical incision and flap management, and bacterial challenge. Five parameters are evaluated:

- Infection signs: 1 point, spontaneous suppuration or on light palpation; 2 points, evident exudate on light palpation; 3 points, slight exudate upon compression of the wound; 4 points, no exudate.
- Hemostasis: 1 point, spontaneous bleeding; 2 points, bleeding at the incision margin with provocation; 3 points, presence of fibrin at the incision margin; 4 points, absence of fibrin at closed incision margin.
- Tissue perfusion (blood supply): 1 point, entire flap necrosis, 2 points, partial soft tissue necrosis at the critical site, e.g., bone graft placement site, 3 points, necrosis at the focal, non-critical site, e.g., adjacent to a tooth; 4 points, no necrotic tissue, and appearance of adequate perfusion.
- Tissue color as a sign of inflammation: 1 point, significant discoloration, e.g., redness; 2 points, moderate discoloration; 3 points, slight discoloration; 4 points, normal appearance as adjacent tissue.
- Epithelialization: 1 point, absent; 2 points, partially present; 3 points, fusion of the wound surface but with evidence of wound surface depression; 4 points, complete fusion of the wound surface by epithelium without depression.

#### 2.1.4. Subject domain.

This is related to patient-reported outcome evaluation, including

- Discomfort: Changes in pain levels were assessed using a scale from 1 to 4, where 1 point, severe; 2 points, moderate; 3 points, slight; 4 points, absent.
- Habit change/function impairment: Changes in habits were evaluated, specifically medication use (including ibuprofen and other medications), as well as functional changes, such as any loss of function, at multiple time points following surgery for both the test and control Groups. The scoring system for medication use was as follows: 1 point for severe use, indicating the need for additional medications beyond the allotted daily dose; 2 points for moderate use, defined as taking the prescribed daily medication dose; 3 points for slight use, characterized by taking half or less of the indicated dose; and 4 points for no use of medications. For the loss of function scale, the scoring was as follows: 1 point for severe impairment; 2 points for moderate impairment; 3 points for slight impairment; and 4 points for no loss of function.

### 2.2. Population and settings

We conducted a retrospective analysis of the healing outcomes of 20 patients who underwent guided bone regeneration (GBR) due to 1-3 mm lateral bone deficiency in the posterior mandible with 1-2 adjacent missing teeth. The study was approved by the University of Michigan Medical School Institutional Review Board (IRB) (HUM00161016) in accordance with the Helsinki Declaration of 1975, as revised in 2013. An informed consent was obtained from all participants before their inclusion in the study. The study design was a randomized controlled trial involving using an amnion-chorion membrane (BioXclude, Snoasis Medical, USA) for the open wound concept as the test group, compared to the closed wound approach using a collagen membrane (ORAMEM, Salvin, USA). Clinical intraoral photography was taken to record the healing progress of patients during four follow-up appointments, which occurred at 3 days, 10 days, 21 days, and 5 months, respectively, resulting in a total of 80 follow-up visits. All patients were treated with 70:30 mineralized and demineralized allograft bone substitutes (Maxxeus, Dayton, OH, USA).

### 2.3. Measurements and outcome projection

Two independent examiners (AR and LM) evaluated the 3 standard intraoral photos taken on the occlusal, facial, and lingual sides at each time point for each wound, totaling 80 follow-up visits for the Biomechanical (1 parameter), Aesthetics (2 parameters), Pathophysiological (5 parameters) Domain’s evaluations. Subject-related outcomes were interpreted from the surveys given to the patients for the Subjective Domain (2 parameters). At each visit, the overall score per each domain was averaged (as shown in Table 1 and Fig 1). A summary score of each wound was calculated by averaging the overall scores of the 4 Domains. Intra-examiner agreement was evaluated by measuring 3 cases twice 15 days apart for memory washout. The MAPS score and the score from each Domain was tested against the 5-month CBCT bone changes to evaluate its predictability. Pre-op and 5-month CBCT images were superimposed using the common references with software (Romexis, Planmeca Oy, Helsinki, Finland). Bone changes at 1-, 3- and 5-mm levels from the edentulous crest were measured with the built-in caliper to 0.01 mm by another masked investigator, a maxillofacial radiologist (NZ), as shown in Table 2.

**Table 2:** Horizontal bone gain outcome measurements using Cone Beam Computed Tomography (CBCT).

Case number	Bone gain		
	@1mm	@3mm	@5mm
1	-1.620	0	0
2	-0.270	0.84	1.43
3	1.620	2.97	3.51
4	2.700	2.43	2.430
5	-5.960	-1.08	-0.81
6	2.210	2.71	1.07
7	0.270	7.31	5.96
8	0.000	1.35	1.62
9	-0.170	2.34	2.35
10	-6.480	-4.86	-1.35
11	-1.340	-1.07	0.58
12	-1.450	-0.86	-0.01
13	2.700	4.05	2.7
14	2.160	3.780	3.24
15	1.080	2.16	0
16	3.520	4.06	3.25
17	-3.250	-1.07	-0.270
18	0.810	0.81	-0.270
19	-0.850	0.59	1.64
20	1.620	2.97	1.080

### 2.4. Statistical analysis

Cohen Kappa’s correlation coefficient was used to evaluate the intra-examiner and inter-examiner agreement for categorical variables with a 95% confidence interval. Inter-examiner agreement was calculated as an overall MAPS score and per individual parameters of the score to evaluate inter-examiner and intra-examiner reliability. A substantial agreement is present when values ranged from 0.6-08, and perfect agreement ranges between 0.8-1. The mean MAPS score was plotted against the time (3, 10, and 21 days and 5 months) and compared with one-way ANOVA to see the changes over time. Linear regression analyses were performed to correlate the MAPS score with radiographic bone changes. A two-tailed p-value of less than 0.05 was considered statistically significant for all tests. Statistical analysis was performed using Graph Pad Prism and R version 4.2.1.

## 3. Results

**Fig 2:**
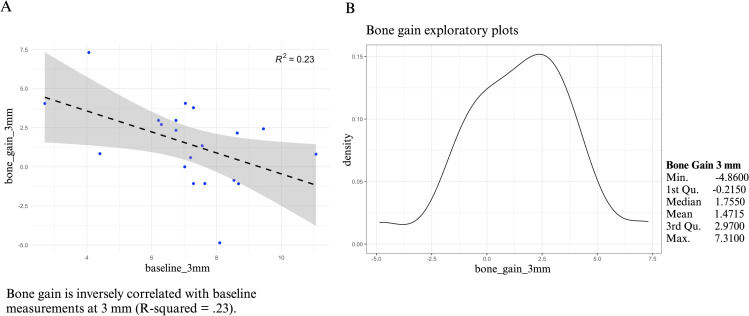
Pearson’s correlation between bone gain at 3 mm below the edentulous crest (A). Bone gains exploratory plots show approximately normally distributed (B).

The patients had a mean age of 58 years (range 27 to 83), with 3 men (15%) and 17 women (85%). The surgical procedure was performed in the posterior mandible (Right/left side ratio 11(55%)/9 cases (45%). Collagen membranes were used in 10 cases (50%), whereas amnion-chorion membranes (BioXclude, Snoasis Medical LLC, USA) were used in 10 cases (50%). The mean CBCT bone changes were 0.14 mm (SD = 2.63 mm, range -6.48 to 3.52 mm), 1.50 mm (SD = 2.54 mm, range -4.86 to 7.31 mm), and 1.41 mm (SD = 1.74 mm, range -1.35 to 5.96 mm) at 1-, 3-, and 5-mm levels from the bone crest, respectively. Pearson’s correlation showed an inverse correlation between baseline bone width and the bone changes at a 3 mm level (R^2^ =  0.23) (Fig 2). The mean CBCT bone gain for the test group at the 1 mm level was -1.86 mm (SD =  2.58, range: -3.25 mm to 0.27 mm), at the 3 mm level was 0.52 mm (SD =  4.14, range: -1.08 mm to 7.31 mm), and at the 5 mm level was 0.95 mm (SD =  2.09, range: -0.135 mm to 5.96 mm). In comparison, the mean CBCT bone gain for the control group at the 1 mm level was 0.77 mm (SD =  2.77, range: -1.45 mm to 3.52 mm), at the 3 mm level was 2.43 mm (SD =  1.58, range: -0.86 mm to 4.05 mm), and at the 5 mm level was 1.67 mm (SD =  1.60, range: -0.81 mm to 3.51 mm). Additionally, a statistically significant difference in bone gain was observed at the 1 mm level between the test and control groups (p =  0.04). However, no significant differences were found in bone gain at the 3 mm and 5 mm levels between the two groups (p =  0.21 and p =  0.40, respectively).

Regarding the “S” subject domain, while the test and control groups initially differed in pain levels and ibuprofen usage during the early post-operative phase, with the test group using more ibuprofen on Days 1 and 2 (P =  0.04), these differences diminished over time. Additionally, no significant differences were observed in the use of other medications between the two groups (p >  0.05).

### 3.1. Intra- and inter-examiner agreement

Cohen’s Kappa values for Examiner 1 and 2 showed intra-examiner agreement of 0.94 (Minimal =  0.74 and Maximal =  1.00) and 0.66 (Minimal =  0.38 and Maximal =  1.00), respectively (Table 3). Inter-examiner agreement for the parameters in the 4 Domains ranged from 0.77 to 1.0. (Tables 4 and 5).

**Table 3:** Intra-examiner agreement summary statistics.

Intra-examiner	Examiner 1		Examiner 2	
	Percent agreement	Cohen’s Kappa	Percent agreement	Cohen’s Kappa
Min	83.33333	0.7377049	58.33333	0.3814433
1^st^ Quartile	93.75000	0.9086294	66.66667	0.5042151
Mean	96.66667	0.9363246	78.33333	0.6598817
3^rd^ Quartile	100.00000	1.0000000	89.58333	0.7931728
Max	100.00000	1.0000000	100.00000	1.0000000

*Cohen’s Kappa agreement was performed with a 95% confidence interval.

**Table 4:** Inter-examiner agreement summary statistics.

	Percent agreement	Cohen’s Kappa
Min	83.54430	0.7696018
1^st^ Quartile	90.18987	0.8475915
Mean	92.65823	0.8850584
3^rd^ Quartile	95.56962	0.9309115
Max	100.00000	1.0000000

*Cohen’s Kappa agreement was performed with a 95% confidence interval.

**Table 5:** Inter-reader agreement of each score.

Individual score parameter	Agreement	Kappa	Lover CI	Upper CI
M_wound edge approximation	96.20253	0.9432811	0.7992021	1.0873601
A_soft tissue quality	91.13924	0.8753241	0.7376512	1.0129970
A_ soft tissue quantity	83.54430	0.7696018	0.6329507	0.9062529
P_haemostasis	93.67089	0.8938029	0.7338247	1.0537811
P_tissue perfusion	93.67089	0.8745036	0.7148037	1.0342034
P_complete wound epithelialization	89.87342	0.8422366	0.6986631	0.9858102
P_signs of infection	92.40506	0.8636560	0.7103475	1.0169644
P_tissue color	86.07595	0.7881779	0.6390910	0.9372649
S_discomfort	100.00000	1.0000000	0.8348031	1.1651969
S_habit change	100.00000	1.0000000	0.8513422	1.1486578

*Cohen’s Kappa agreement was performed with a 95% confidence interval.

### 3.2. MAPS score changes overtime

**Fig 3:**
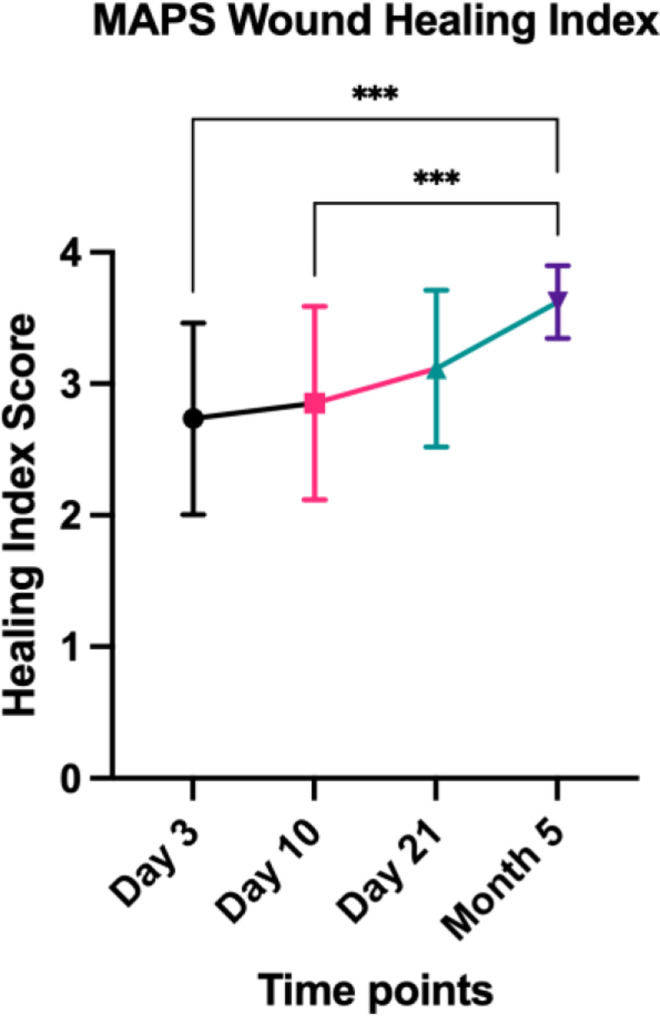
MAPS score changes over time at 3-, 10-, 21 days, and 5 months.

**Fig 4:**
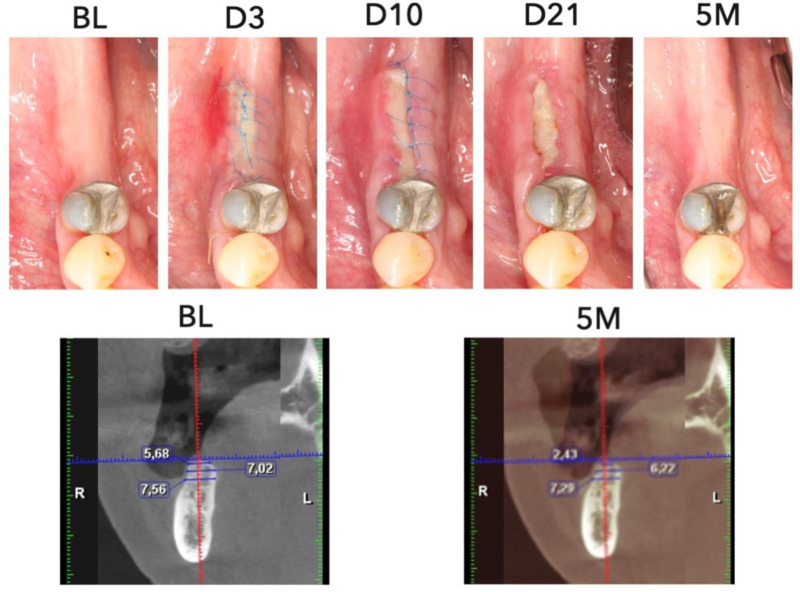
Case demonstration of compromised healing after guided bone regeneration procedure. Top row shows clinical intraoral photography from baseline (BL), 3 days (D3), 10 days (10D), 21 days (D21), and 5 months (5M). Bottom row shows CBCT bone width measurements at baseline (BL), 5 months (5M). There is evidence of inferior bone gain seen in clinical photos over time due to compromised healing and shown in bone loss measurements in the follow-up CBCT.

**Fig 5:**
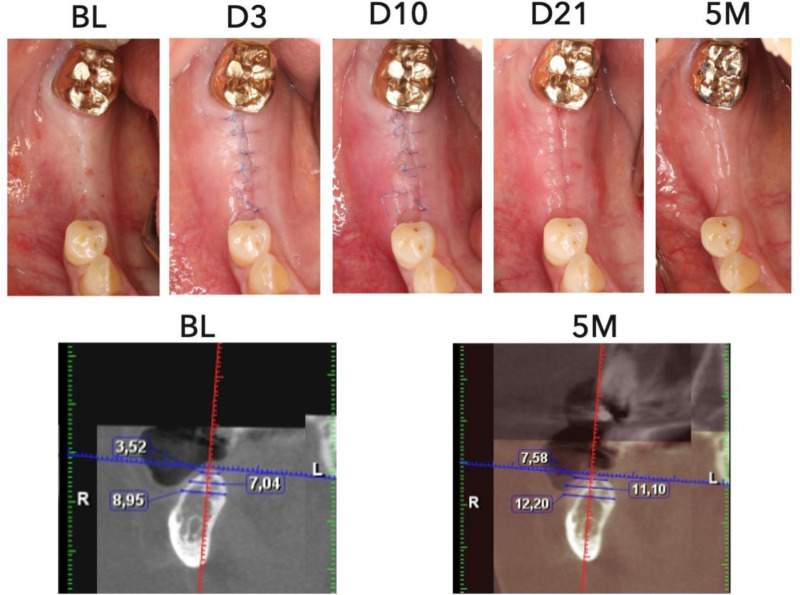
Case demonstration of favorable healing after guided bone regeneration procedure. Top row shows clinical intraoral photography from baseline (BL), 3 days (D3), 10 days (10D), 21 days (D21), and 5 months (5M). Bottom row shows CBCT bone width measurements at baseline (BL), 5 months (5M). There is clear evidence of superior bone gain seen in clinical photos over time due to optimal healing and shown in bone gain measurements in the follow-up CBCT.

**Fig 6:**
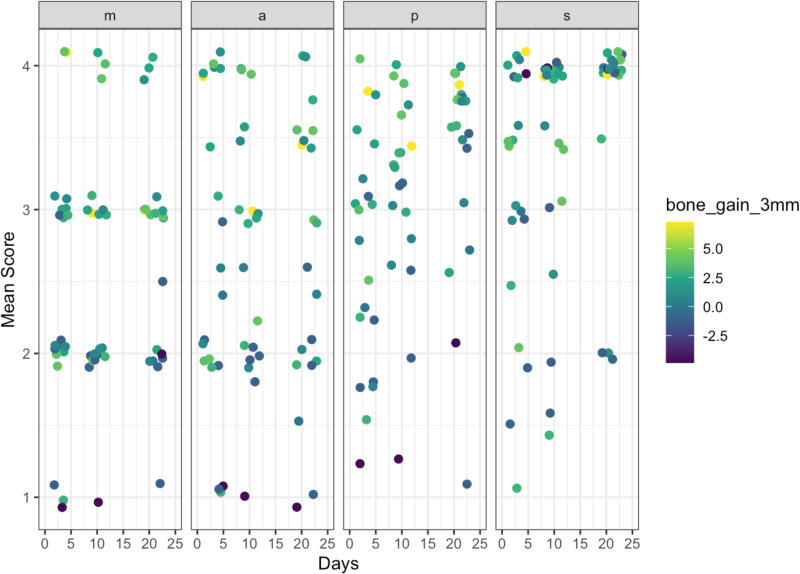
Scatterplot of mean MAPS scores with bone gain at 3 mm at different time points. The color scale bar indicates bone gain, where dark blue can be seen as negative bone gain (up to -2.5 mm), light blue no gain ( ~ 0 mm), dark green mild bone gain (up to 2.5 mm), light green moderate bone gain (up to 5 mm), and yellow high bone gain (>5 mm). Higher M,A, P at any time point is associated with higher bone gain.

The mean score was 2.7 (SD = 0.7), 2.7 (SD = 0.6), 3.1 (SD = 0.6), and 3.6 (SD = 0.3) at 3-, 10-, and 21-day, and 5-month visits, respectively, suggesting an overall resolution of inflammation and improvement in soft tissue tone. The scores between 3-day and 5-month visits and 10-day and 5-month visits were significantly different (p < 0.0001) (Fig 3). Figs 4 and 5 showed examples of compromised and favorable healing with clinical occlusal photos and baseline as well as 5-month CBCT images. In addition, a scatterplot of the mean individual MAPS scores parameters (of both examiners) over time (up to day 21) is presented. The color scale bar indicates bone gain, where dark blue can be seen as negative bone gain (up to -2.5 mm), light blue no gain ( ~ 0 mm), dark green mild bone gain (up to 2.5 mm), light green moderate bone gain (up to 5 mm), and yellow high bone gain (>5 mm). Preliminary results indicate that higher M, A, and P values at any time point were associated with higher bone gain (Fig 6). The mean MAPS scores for the test group at the 3-day, 10-day, 21-day, and 5-month visits were 2.8 (SD =  0.8), 2.7 (SD =  0.6), 2.7 (SD =  0.6), and 3.5 (SD =  0.3), respectively. For the control group, the mean MAPS scores at the same time points were 2.7 (SD =  0.6), 3.1 (SD =  0.6), 3.5 (SD =  0.4), and 3.8 (SD =  0.2), respectively.

When comparing the test and control groups, no statistically significant differences were found in the overall MAPS scores at the 3-day and 10-day visits (p =  0.70, SD =  0.73; p =  0.14, SD =  0.61, respectively). However, a statistically significant difference was observed at the 21-day and 5-month visits (p =  0.001, SD =  0.60; p =  0.06, SD =  -0.28, respectively). Specifically, the control group showed higher overall MAPS scores than the test group at both 21 days (3.5 vs. 2.7, respectively) and 5 months (3.8 vs. 3.5, respectively).

### 3.3. MAPS score vs. radiographic bone changes

**Fig 7:**
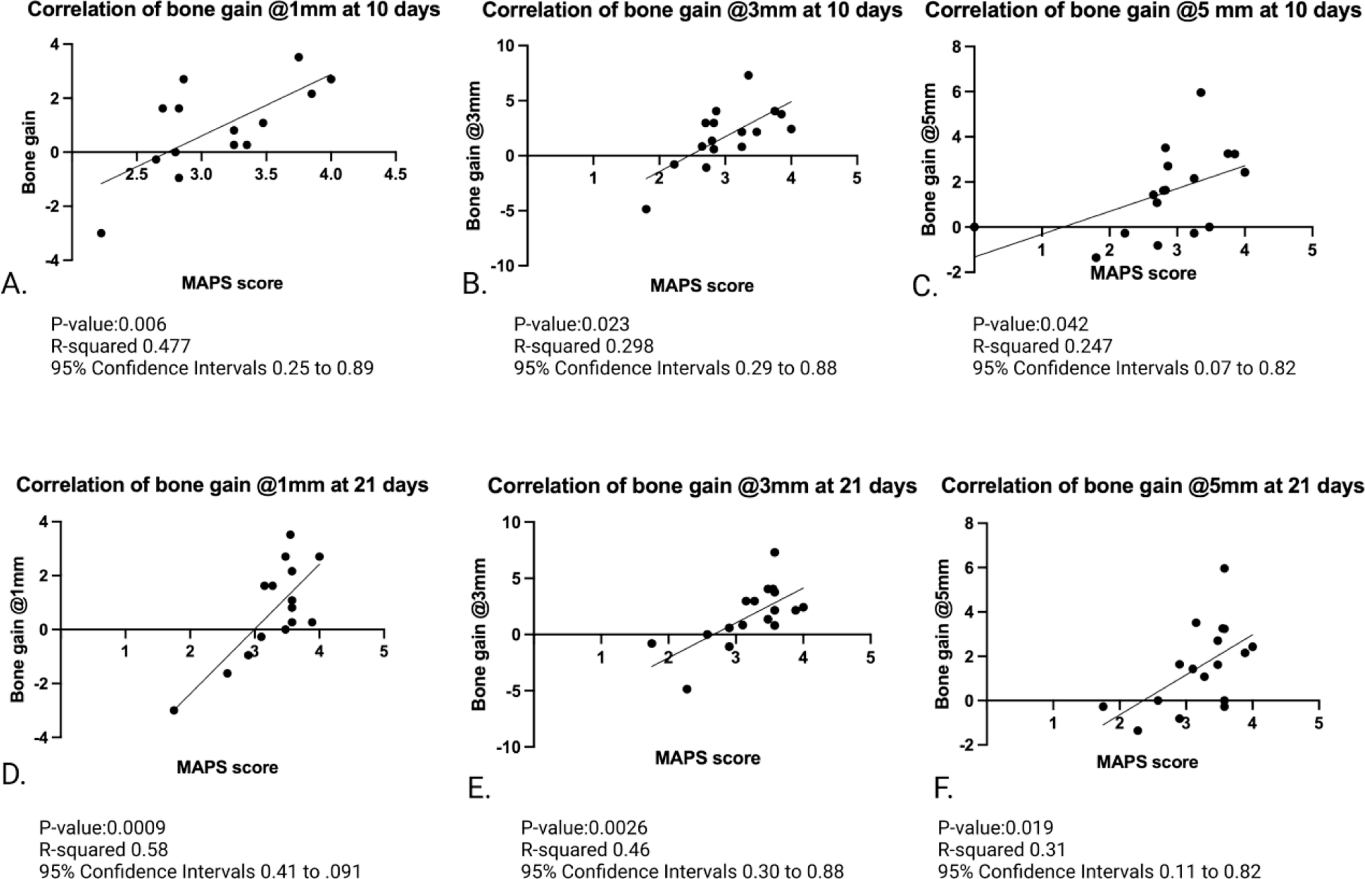
Multiple Pearson’s correlations between bone gain and MAPS scores at 1, 3, and 5 mm at 10 days (A-C) and 21 days (D-F). There is statistical significance of bone gain at 1, 3, and 5 mm at 10 and 21 days when compared to the MAPS score (p-value <  0.05). MAPS score correlated with bone changes after GBR procedures, indicating its potential for estimating hard tissue regenerative outcomes.

Because of the high inter-observer agreement, the MAPS scores were averaged from 2 observers to plot against the radiographic bone changes. Statistically significant correlations were found between the 3-day mean score and 3-mm bone gain (R^2^ =  0.7, p =  0.01), 10-day mean score and bone gain at 1 mm (R^2^ =  0.47, p =  0.006,), 3 mm (R^2^ =  0.29, p =  0.02,), and 5 mm (R^2^ =  0.24, p = 0.042) levels. The correlations of 21-day mean score and bone gain were also statistically significant at 1 mm (R^2^ =  0.58, p =  0.0009,), 3 mm (R^2^ =  0.46, p =  0.0026,), and 5 mm (R^2^ =  0.31, p =  0.019) (Fig 7).

**Fig 8:**
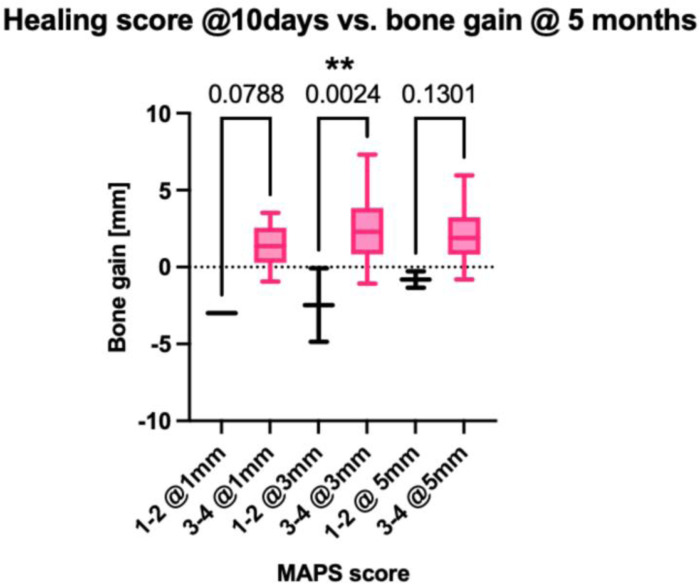
Lower (scores of 1-2), and higher (scores of 3-4) MAPS scores were correlated with bone gain measured in CBCT at 1, 3, and 5 mm. There is statistical significance for bone gain at 3 mm when correlated with the grouped low and high MAPS scores (p-value <  0.005).

Furthermore, the bone gain at 1, 3, and 5 mm were compared between the combined MAPS scores of 1-2 (lower MAPS) and 3-4 (higher MAPS) at 3, 10, and 21 days. At day 10, the 3-mm bone gain was significantly more in the higher MAPS (mean =  3.29 mm, SD =  1.84 mm) than in the lower MAPS group (mean =  0.02 mm, SD =  2.32 mm, p = 0.002). (Fig 8). Additionally, when analyzing the MAPS scores versus radiographic bone gain in both the test and control groups, the findings showed that in the test group, the correlations between the mean MAPS scores at 3 days, 10 days, 21 days, and 5 months, and bone gain at the 1-, 3-, and 5-mm levels were statistically significant (p <  0.05 at all levels and all follow-up time points). In contrast, in the control group, the correlations between the 3-day mean MAPS score and bone gain at the 3- and 5-mm levels did not show statistically significant differences (p =  0.37 and p =  0.11, respectively). However, in the control group, statistically significant correlations were found between the 3-day mean MAPS score and bone gain at the 1-mm level, as well as between the 10-day, 21-day, and 5-month mean MAPS scores and bone gain at the 1-, 3-, and 5-mm levels (p <  0.05 for all).

### 3.4. Statistical modeling

Linear regression models were used to identify the time point (3, 10, 21 days, and 5 months) that fit the best with the 3-mm bone gain, which is clinically relevant in this indication, compared to 1-mm and 3-mm levels. The 10-day MAPS score turns out the most predictive of bone gain, with the lowest RMSE (1.32) and highest R^2^ (0.75), calculated by the method of leave-one-out-cross-validation (Table 6). Next, a stepwise procedure was used to perform variable selection. The best model, selected via AIC contained only the average A score, average P score, and baseline bone width. Regression coefficients and 95% confidence intervals are provided in Table 7. Increasing the average A score by 1 point at 10 days is significantly associated with an increase in bone gain of 1.6 (95% CI: 0.54-2.66, p = .006). In addition, increasing the average P score by 1 point at 10 days is associated with an increase in bone gain of 1.23 (95% CI: -0.04-2.51, p = .057). Finally, increasing the baseline 3mm-bone width by 1 mm is associated with a decrease in bone gain of 0.76 mm (95% CI -1.07 to 0.46, p < .001) (Table 8).

**Table 6:** Fit linear model at each time point with an individual average score of M, A, P, and from baseline to 5 months follow up at 3 mm level bone gain.

RMSE	R-squared	MAE	Model
2.636108	0.1286608	2.115492	3 days
1.321189	0.7465901	1.032047	10 days
1.675164	0.5994803	1.336843	21 days
2.225491	0.2980006	1.772055	5 months

*The leave-one-out cross-validation method was used to calculate the root mean square error (RMSE) and R-squared.

**Table 7:** Fit Model Summary with dependent variables of the individual average score of M, A, P, and from baseline to 5 months follow up at 3 mm level bone gain.

Dependent variable			
Predictors	Estimates	CI	p value
(Intercept)	-2.41	-7.00 –2.18	0.278
A	1.08	-1.10 – 3.25	0.303
M	0.47	-1.53 – 2.46	0.621
P	1.34	-0.01 – 2.69	0.052
S	0.26	-0.39 – 0.92	0.398
3 mm (BL- 5MO)	-0.73	-1.06 – -0.41	<0.001

*R^2^/ R^2^ adjusted: 0.87/0.82.

**Table 8:** Final predictive model.

Dependent variable			
Predictors	Estimates	CI	p value
(Intercept)	-1.18	-4.68 –2.32	0.484
A	1.60	0.54 – 2.66	0.006
P	1.23	-0.04 – 2.51	0.057
3 mm (BL- 5MO)	-0.76	-1.07 – -0.46	<0.001

*R^2^/ R^2^ adjusted: 0.86/0.84.

## 4. Discussion

### 4.1. Primary findings

In this study, the MAPS index was evaluated for the healing assessment of 20 patients who had a GBR procedure using 2 types of resorbable membranes for correcting minor (1-3 mm) lateral ridge deficiency. The available data suggested an acceptable reproducibility of this index at both intra-examiner (κ= 0.94 for examiner 1 and κ=0.66 for examiner 2) and inter-examiner levels (κ= 0.89).

The MAPS score shows continuous improvement throughout the 5-month healing period. However, there is no statistically significant difference between the Day 21 and 5-month scores, which reflects the typical clinical healing pattern observed with collagen membranes in GBR (Fig 3). The overall MAPS score correlated with bone changes after GBR procedures, indicating its potential for estimating hard tissue regenerative outcomes (Figs 6 and 7). More specifically, the 10-day seems reliable and practical to predict the healing outcome, based on the fit linear model. Comparisons in bone changes were made between MAPS scores of 1-2 and 3-4, showing at the 10-day follow-up, normal/accelerated healing scores showed a statistically significant bone gain compared to compromised/delayed healing scores (Fig 8). Furthermore, of the 4 Domains that contribute to the MAPS score, the stepwise analyses suggest the Aesthetic/Anatomical (A) and Pathophysiological Domains (P) correlate with 5-month bone changes (Tables 7 and 8). Preliminary evidence indicates early soft tissue healing related to inflammation status and tissue features may predict final hard tissue healing outcomes [1–3,6,11]. The biomechanical and subject-related domains, even not correlated with the outcome, should be carefully evaluated as an integral part of wound healing evaluation. Additionally, the mean CBCT bone gain for the test group showed bone loss of -1.86 mm at the 1-mm level, compared to a marginal gain of 0.77 mm in the control group, which was statistically significant. No significant differences were observed at the 3-mm and 5-mm levels. However, the control group exhibited higher bone gain at the 1-, 3-, and 5-mm levels compared to the test group.

For the MAPS scores, no significant differences were found at the 3-day and 10-day visits. However, significant differences were observed at both the 21-day and 5-month visits, with the control group showing higher MAPS scores at both time points (21 days: 3.5 vs. 2.7; 5 months: 3.8 vs. 3.5). When analyzing the correlation between MAPS scores and bone gain, the test group showed lower bone gain and MAPS scores at the 1-mm level compared to the control group. While no significant differences were found at the 3-mm and 5-mm levels, the control group demonstrated higher bone gain at these levels. These results were further supported by the significant differences in MAPS scores at 21 days and 5 months, favoring the control group.

### 4.2. Inclusion of the current literature and emerging technology

The MAPS Index encompasses the key parameters that was included in the existing indices [2,3,11,12,7–18,20–22]. These parameters are categorized into the 4 domains that are closely tied to the sequences of wound healing, soft tissue changes, and patient-centered outcomes. Previous healing indexes examine the patient’s perception verbally and wound appearance “visually” [18–22]. Emerging evidence shows biomechanical homeostasis relies on a balance between the suture tension, flap contraction from internal stress, swelling and muscle pulling during function, and biomaterial stability [2,14,15,22,52,53]. Imbalance in this intricate system may cause wound edge opening, increased risk of infection, and compromised outcome. Early biological events, e.g., tissue reperfusion, inflammation, and epithelialization maintain and sustain uneventful wound healing [6,10,14,54,55]. A failure in tissue perfusion and epithelialization and uncontrolled inflammatory responses could lead to wound instability and inferior outcome. Therefore, monitoring the clinical representations of these critical healing events and evaluating the degree of stability by not only visual examination but also functional evaluation by gentle palpation, compression, provocation, and pull of the wound could lead to more useful healing information. CBCT has been validated in the literature for its clinically acceptable accuracy in bone measurements [23,56–61]. Emerging imaging methods, e.g., ultrasound can evaluate the wound edge in cross-sectional view, quantify blood flow, and tissue elasticity, etc., and laser speckle for superficial blood flow, thus can potentially provide objective and quantifiable measurements of wound healing status [16,28,62–64].

### 4.3. Versatile in evaluation timing and purposes

This index could be a useful tool for clinicians to document wound healing status at different time points for various surgical procedures, e.g., periodontal tissue regeneration, soft tissue plastic surgery, ridge augmentation, etc. Previous indices mainly focus on wound healing at specific time points, e.g., 1-2 weeks [22] and for evaluation of a certain procedure. The MAPS evaluates fundamental wound healing components and therefore envisions accommodating assessment of oral wound healing from different types of procedures. Together the individual scores of A, and P with baseline measurements explain about 77% of the variability in bone changes (using the cross-validated estimate). It is also an aim of MAPS to guide the check-up frequency, selection of follow-up procedures, and assignment of treatment outcomes/prognosis. Each domain has different focuses and evaluations, potentially leading to the identification of a specific failing component within the complicated healing system.

### 4.4. Research indications

These preliminary data suggest a potential use of this MAPS index for systematically evaluating wound healing after GBR and predicting hard tissue healing outcome. Based on this framework, this index system can be further validated by including a larger group of representative examiners with mixed surgical experiences. A prospective study can be designed to test the predictability of this system for not only hard tissue regeneration of various defect sizes but also soft tissue reconstructive procedures for non-esthetic as well as esthetic indications.

### 4.5. Clinical applications

The MAPS index provides clinicians with a comprehensive tool to assess and monitor wound healing outcomes, considering the key components of biomechanical, aesthetic/anatomical, pathophysiological, and subject-related variables. This proposal could be simplified by giving a summary score for each domain. This standardized approach to assessing wound healing outcomes could enable clinicians to deliver more effective and personalized would monitoring protocol, once validated by larger-scale studies.

## 5. Study limitations

The results of this study are based on a specific surgical indication, i.e., minor lateral ridge augmentation, relatively small sample size, a retrospective evaluation of wound healing by reviewing clinical photos and patients’ feedback. External validation is a crucial next step to assess the prediction accuracy of the MAPS scores. We acknowledge that some parameters are currently subjective. In clinical settings, where efficiency is critical, a certain level of subjectivity may be unavoidable. However, tissue changes can be objectively quantified for research purposes using intraoral scanners, ensuring greater reproducibility and precision.

## 6. Conclusions

The MAPS Index was introduced, encompassing the biomechanical, aesthetic/anatomical, pathophysiologic, and subject-related domains that are separately evaluated and yet collectively considered. Preliminary data show its potential of correlating bone augmentation outcome and usefulness for further understanding of the wound healing steps and guidance of wound management to benefit patients’ welfare.
